# Factors Associated with Atrial Fibrillation in Heart Failure with Preserved and Mildly Reduced Ejection Fraction: A Real-World Cohort Study

**DOI:** 10.3390/jcm15072747

**Published:** 2026-04-05

**Authors:** Milen Minchev, Ivan Gruev, Stefan Naydenov

**Affiliations:** 1 Clinic of Cardiology, National Multi-Profile Transport Hospital Tsar Boris III—Sofia, 1220 Sofia, Bulgaria; milminchev@yahoo.com (M.M.); ivangruev@yahoo.com (I.G.); 2 Department of Internal Diseases “Prof. St. Kirkovich”, Medical University of Sofia, 1431 Sofia, Bulgaria

**Keywords:** atrial fibrillation, heart failure, HFpEF, HFmrEF, echocardiography, risk prediction

## Abstract

**Background:** Atrial fibrillation (AF) frequently coexists with heart failure (HF) and worsens clinical outcomes. However, factors associated with AF in HF with preserved (HFpEF) and mildly reduced ejection fraction (HFmrEF) remain poorly defined. This study aimed to identify clinical, laboratory, and echocardiographic determinants of AF in these HF phenotypes. **Methods:** This retrospective single-center observational study included 700 consecutive patients with HF hospitalized between January 2018 and December 2023. The median age was 74 years (IQR 66–80). Women predominated in the cohort (55.3% vs. 44.7%, *p* < 0.001). Based on echocardiographically assessed left ventricular ejection fraction, patients were stratified into groups with preserved (≥50%), mildly reduced (41–49%), and reduced (≤40%) ejection fraction. Determinants of AF were evaluated using univariate and multivariate logistic regression analyses, and model discrimination was assessed using ROC analysis. **Results:** Strongest determinants of AF in our patients with HFpEF and HFmrEF were left atrial size (OR 1.114 per mm increase; 95% CI 1.054–1.177; *p* < 0.001), moderate and severe tricuspid regurgitation (OR 4.092; 95% CI 1.977–8.466; *p* < 0.001 and OR 6.957; 95% CI 2.482–19.499; *p* < 0.001), male gender (OR 1.680; 95% CI 1.076–2.621; *p* = 0.022) and advanced age (OR 1.070 per year; 95% CI 1.032–1.109; *p* < 0.001). **Conclusions:** In patients with HFpEF and HFmrEF, AF is strongly associated with atrial remodeling, with left atrial enlargement as the key structural determinant. The identified associations may contribute to an improved understanding of AF in HFpEF and HFmrEF; however, their potential role in risk stratification requires validation in prospective studies.

## 1. Introduction

Atrial fibrillation (AF) is the most common sustained cardiac arrhythmia worldwide, affecting approximately 2–3% of the general population, with prevalence increasing markedly with age [1,2,3,4,5]. Epidemiological studies indicate that the lifetime risk of AF exceeds 30% in individuals older than 55 years [1,2,3]. According to data from the Global Burden of Disease project, more than 59 million people worldwide are currently living with AF, and its prevalence is expected to continue rising due to population aging, improved survival from cardiovascular diseases, and enhanced detection of asymptomatic cases [3,6]. AF carries substantial clinical significance, as it is associated with an increased risk of ischemic stroke, systemic thromboembolism, heart failure (HF), cognitive impairment, and cardiovascular mortality [1,3,7].

Heart failure represents another major global health challenge, characterized by high morbidity, mortality, and healthcare burden [8,9,10,11]. The prevalence of HF is estimated at approximately 1–2% in the adult population and exceeds 10% among individuals older than 70 years [8,9,10,11].

AF and HF frequently coexist and share complex bidirectional interactions [12,13,14]. AF may precipitate or worsen HF through loss of atrial contribution to ventricular filling, irregular ventricular rhythm, and tachycardia-induced cardiomyopathy [1,12,14,15]. Conversely, structural and hemodynamic changes associated with HF—including elevated filling pressures, atrial dilation, myocardial fibrosis, and neurohormonal activation—create a substrate that promotes atrial electrical instability and facilitates the development and maintenance of AF [12,13,14,15].

Particular interest has recently focused on patients with HF with preserved (HFpEF) and mildly reduced ejection fraction (HFmrEF), which represent a rapidly expanding and heterogeneous HF population [9,16,17,18]. In these phenotypes, AF is the most common arrhythmia, with reported prevalence ranging between 15% and 40% in various clinical studies and registries [13,19,20,21,22]. The coexistence of AF and HFpEF/HFmrEF is associated with more severe symptoms, higher rates of hospitalization, and worse long-term prognosis compared with patients in sinus rhythm [13,19,20,21,22].

Multiple factors have been implicated as potential determinants of AF in patients with HF, including advanced age, male sex, hypertension, obesity, diabetes mellitus, chronic kidney disease, and structural heart disease [23,24,25,26,27]. In addition, electrocardiographic abnormalities and echocardiographic markers of atrial remodeling—particularly left atrial (LA) enlargement—have been consistently associated with increased AF risk [23,24,27,28]. Biomarkers reflecting myocardial stress, inflammation, and neurohormonal activation have also been proposed as additional contributors to AF development [29,30,31,32]. However, available data remain heterogeneous, and many studies have focused predominantly on HF with reduced ejection fraction (HFrEF), leaving important gaps in the understanding of factors associated with AF specifically in HFpEF and HFmrEF populations [13,21,28,32].

Identification of reliable determinants of AF occurrence in patients with HFpEF and HFmrEF is therefore of considerable clinical importance. Early recognition of individuals at increased risk may facilitate targeted screening strategies, earlier diagnosis of AF, and more effective risk stratification and management. Therefore, the aim of the present study was to identify clinical, laboratory, and echocardiographic factors associated with AF in patients with HFpEF and HFmrEF.

## 2. Materials and Methods

### 2.1. Study Design and Population

This retrospective single-center observational study included 700 consecutive patients with chronic HF hospitalized in a tertiary cardiology clinic between January 2018 and December 2023. The diagnosis of chronic HF was established in accordance with European Society of Cardiology (ESC) guidelines, based on clinical presentation, laboratory findings, and echocardiographic criteria [8]. In patients with preserved (≥50%) left ventricular ejection fraction (LVEF), the diagnosis of HFpEF was established according to the 2021 ESC heart failure guidelines, requiring the presence of typical symptoms and signs of HF, elevated natriuretic peptide levels, and objective evidence of relevant structural and/or functional cardiac abnormalities, e.g., LA enlargement, left ventricular (LV) hypertrophy, or diastolic dysfunction [8].

The median age of the study population was 74 years (interquartile range [IQR] 66–80 years), and women predominated (55.3% vs. 44.7%, *p* < 0.001).

Patients were stratified according to left ventricular ejection fraction (LVEF), assessed by transthoracic echocardiography, into the following HF phenotypes: HF with preserved ejection fraction (LVEF ≥ 50%), HF with mildly reduced ejection fraction (LVEF 41–49%), and HF with reduced ejection fraction (LVEF ≤ 40%), in accordance with the current ESC guidelines [8]. HFpEF was the most prevalent phenotype (487 patients, 69.6%), followed by HFrEF (107 patients, 15.3%) and HFmrEF (106 patients, 15.1%) (*p* < 0.001).

In addition to HF phenotype classification, patients were further categorized according to cardiac rhythm. For the purposes of the analysis, all clinical forms of AF—including paroxysmal, persistent, long-standing persistent, and permanent AF—were considered collectively as a single entity, defined as the presence or absence of AF. Accordingly, patients were classified into two groups: sinus rhythm (*n* = 317, 45.3%) and AF (*n* = 383, 54.7%), encompassing all AF phenotypes as defined by current ESC guidelines [1]. AF was significantly more prevalent in the study population (*p* = 0.013).

Clinical data including demographic characteristics, comorbidities, cardiovascular risk factors, medication history, physical examination findings, laboratory results, and instrumental investigations were collected retrospectively from hospital discharge summaries and available medical records and entered into a standardized data collection form.

Due to the retrospective design and lack of longitudinal follow-up, the analyzed variables should be interpreted as factors associated with prevalent atrial fibrillation rather than predictors of incident atrial fibrillation.

### 2.2. Inclusion and Exclusion Criteria

Patients were eligible for inclusion if they met the following criteria:

- age ≥18 years;
- documented diagnosis of chronic HF according to ESC HF guidelines [8,9];
- instrumental assessment of LVEF within the previous 12 months;
- documented cardiac rhythm (sinus rhythm, AF, or other rhythm) confirmed by electrocardiography (ECG) or Holter ECG monitoring;
- availability of sufficiently complete clinical, laboratory, and imaging data required for the analysis.

Patients were excluded if they had:

- an acute cardiovascular event (myocardial infarction, ischemic or hemorrhagic stroke, acute peripheral arterial incident, or other) ≤90 days prior to the time of assessment;
- implanted rhythm-modifying cardiac devices (e.g., permanent pacemakers, implantable cardioverter-defibrillators, or cardiac resynchronization therapy devices);
- significant uncontrolled comorbidities that could influence the analyzed parameters (e.g., active malignancy, severe renal failure, uncontrolled endocrine disorders);
- incomplete clinical, laboratory, or echocardiographic data required for the analysis.

Accordingly, a complete-case analysis approach was applied. A total of 94 patients were excluded due to incomplete data.

### 2.3. Electrocardiography and Holter ECG Monitoring

A standard 12-lead ECG was performed in all patients using a Mortara ELI 280c electrocardiograph. ECG recordings were analyzed for heart rhythm, heart rate, electrical axis, P-wave morphology, PR interval, QRS duration and morphology, as well as ST-segment and T-wave abnormalities.

When clinically indicated, 24–72-h Holter ECG monitoring was performed using 3-channel (Contec V5.5.2.3, Contec Medical Systems Co., Ltd., Qinhuangdao, China) or 7-channel (Medilog Darwin V2, Schiller Ltd., Baar, Switzerland) devices to detect rhythm disturbances and confirm the presence of AF. The diagnosis of this rhythm disorder was established based on electrocardiographic findings in accordance with current ESC guidelines [1]. Both previously diagnosed AF and AF detected during hospitalization were included in the analysis.

Patients with previously documented AF and those with newly identified AF during hospitalization were analyzed together as a single group. Holter ECG monitoring was performed only when clinically indicated; therefore, asymptomatic or paroxysmal AF episodes may have been underdetected.

### 2.4. Echocardiographic Assessment

All patients underwent transthoracic echocardiography performed by experienced cardiologists using standard imaging protocols. Examinations were performed using a General Electric Vivid E95 (GE HealthCare Technologies, Inc., Chicago, IL, USA) ultrasound system equipped with a 4Vc matrix-array sector transducer (GE HealthCare Technologies, Inc., Chicago, IL, USA) and were conducted in accordance with the recommendations of the European Association of Cardiovascular Imaging [33].

Standard M-mode and two-dimensional imaging were used for cardiac structural assessment. Left ventricular systolic function was evaluated by calculating LVEF using the biplane Simpson method.

Cardiac chamber dimensions and geometry were assessed by measuring LA size in the parasternal long-axis view and LA longitudinal diameter in the apical four-chamber view, as well as interventricular septal thickness and posterior LV wall thickness. LV size was further characterized by measuring end-diastolic and end-systolic diameters in the parasternal long-axis view and LV end-diastolic and end-systolic volumes in the apical four-chamber view.

### 2.5. Laboratory Investigations

Laboratory analyses were performed at the central clinical laboratory of the hospital where our study was conducted. The analyzed variables included potassium, sodium, hemoglobin, hematocrit, C-reactive protein (CRP), N-terminal pro-B-type natriuretic peptide (NT-proBNP), uric acid, serum creatinine, and estimated glomerular filtration rate (eGFR) calculated using the CKD-EPI 2021 formula [34].

### 2.6. Ethical Considerations

The study protocol was registered in the ClinicalTrials.gov Protocol Registration and Results System (PRS), National Library of Medicine, under the unique protocol ID PK36-3117-27/04/22. It was conducted in accordance with the ethical principles of the Declaration of Helsinki and the principles of Good Clinical Practice. Due to the retrospective and non-interventional design of the study and the use of fully anonymized clinical data, the requirement for informed consent was waived in accordance with applicable national regulations and institutional policies.

### 2.7. Statistical Analysis

All statistical analyses were performed using SPSS software (IBM SPSS Statistics, version 19.0, IBM Corp., Armonk, NY, USA). Continuous variables are presented as median values with interquartile ranges (IQR), while categorical variables are expressed as absolute numbers and percentages. Categorical variables were compared using the χ^2^ test, while continuous variables were compared using the Kruskal–Wallis test or Mann–Whitney U test as appropriate.

Potential factors associated with AF were initially evaluated using univariate logistic regression analysis. Variables for inclusion in the multivariable logistic regression model were selected based on a combination of univariate statistical significance and clinical relevance, taking into account established risk factors reported in the literature.

Multivariable analysis was then performed to identify independent factors associated with AF. To reduce the risk of multicollinearity, correlations among candidate variables were systematically assessed, with no evidence of strong inter-variable correlations identified.

HFpEF and HFmrEF populations were analyzed jointly in the multivariable model, as these phenotypes share overlapping pathophysiological characteristics and were considered as a continuum of non-reduced ejection fraction HF for the purposes of this analysis.

The performance of the model was evaluated using receiver operating characteristic (ROC) curve analysis, with calculation of the area under the curve (AUC). Calibration was not formally assessed using established statistical methods (e.g., calibration plots or Hosmer–Lemeshow test), and the model’s performance should be interpreted in this context.

A *p*-value < 0.05 was considered statistically significant.

Artificial Intelligence Use: Artificial intelligence (ChatGPT, OpenAI, GPT-5.3, 2026) was used solely to assist in the creation of graphical figures included in this article.

## 3. Results

Table 1 summarizes the demographic and epidemiological characteristics of patients according to HF phenotype. HFpEF was the predominant phenotype (69.6%). A significant difference in sex distribution was observed, with male predominance in the HFmrEF and HFrEF groups (*p* < 0.001). Age and age distribution did not differ significantly between phenotypes (*p* = 0.738 and *p* = 0.051), with patients aged ≥70 years constituting the majority of the cohort. The distribution of cases before and after the COVID-19 pandemic was similar across HF phenotypes and did not reach statistical significance.

Table 2 summarizes the clinical profile of the study population, including HF duration, NYHA functional class, and the prevalence and forms of AF according to HF phenotype.

HF duration did not differ significantly between groups (*p* = 0.077).

NYHA class distribution differed significantly (*p* < 0.001), with NYHA III predominating across all phenotypes and NYHA IV being more frequent in HFrEF. The predominance of NYHA III likely reflects the hospitalized nature of the study population, in which patients are typically admitted due to symptomatic decompensation or worsening HF.

Heart rhythm distribution also differed significantly (*p* < 0.001), with sinus rhythm more common in HFpEF, whereas permanent AF predominated in HFmrEF and HFrEF.

Table 3 summarizes the comorbidity profile of HF patients according to HF phenotype. Most cardiovascular and non-cardiovascular comorbidities and cardiometabolic risk factors were similarly distributed across phenotypes. However, coronary artery disease (*p* = 0.009), chronic kidney disease (*p* = 0.015), peripheral arterial disease (*p* = 0.004), and prior cardiac surgery (*p* < 0.001) were significantly more frequent among patients with HFmrEF and HFrEF.

Table 4 presents the distribution of the CHA_2_DS_2_-VA score according to HF phenotype and cardiac rhythm. In patients with HFpEF, the median score ranged from 3 to 4 points across different rhythm categories. Comparable values were observed in patients with HFmrEF and HFrEF. Overall, the CHA_2_DS_2_-VA score showed similar distributions across HF phenotypes, with no statistically significant differences between groups (*p* = 0.568).

Table 5 presents the distribution of the HAS-BLED score according to HF phenotype and cardiac rhythm. Median HAS-BLED values were 2 points across all HF phenotypes, with largely overlapping interquartile ranges. Accordingly, no statistically significant differences were observed between groups (*p* = 0.523).

Table 6 summarizes the hemodynamic and anthropometric characteristics of the study population according to HF phenotype. Office systolic blood pressure (BP) differed significantly between groups (*p* = 0.002), with higher values observed in patients with HFpEF. Heart rate also showed significant differences across phenotypes (*p* < 0.001), with higher values in HFmrEF and HFrEF. No significant differences were observed in diastolic BP or body mass index (BMI).

Table 7 presents selected laboratory parameters of the study population according to HF phenotype. Serum sodium levels and eGFR differed significantly between groups (*p* = 0.038 and *p* = 0.037, respectively). In contrast, potassium levels, hemoglobin concentration, and serum creatinine values were comparable across HF phenotypes.

Table 8 summarizes the echocardiographic characteristics of the study population according to HF phenotype. Significant differences were observed in LA size and multiple LV structural and functional parameters across HF phenotypes (all *p* < 0.01). Patients with HFrEF exhibited larger LV dimensions and volumes, whereas HFpEF was characterized by smaller ventricular size. A progressive increase in LA dimensions was also observed across HF phenotypes, with the largest values detected in patients with HFrEF. These findings reflect distinct patterns of cardiac remodeling across HF phenotypes, with more pronounced ventricular dilation in HFrEF and relatively preserved ventricular geometry in HFpEF.

To identify factors associated with AF across different HF phenotypes, univariate binary logistic regression analyses were performed separately for each subgroup (Table 9, Table 10, Table 11, Table 12, Table 13 and Table 14). In HFpEF, AF was associated with the broadest range of demographic, clinical, laboratory, echocardiographic, valvular, and treatment-related variables. In HFmrEF, the spectrum of significant associations was more limited and mainly included age, heart rate, and LA size. In HFrEF, AF was associated predominantly with age, hemodynamic parameters, and indices of left ventricular geometry. Multivariate logistic regression further identified distinct phenotype-specific independent factors associated with AF.

Based on the identified factors associated with AF, an associative model for AF was developed in the pooled HFpEF/HFmrEF population (*n* = 593). The model incorporated age, sex, heart rate, office systolic BP, eGFR, LA size measured in the parasternal long-axis view, the presence and severity of tricuspid regurgitation, metabolic syndrome, and an indicator variable for HF phenotype. Given the cross-sectional design and lack of internal validation, these findings reflect associations rather than predictive performance.

**Figure 1:**
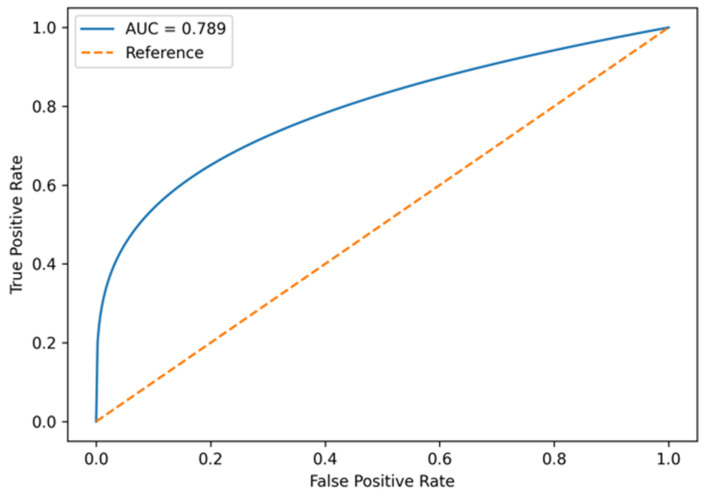
ROC curve of the AF associative model in patients with HFpEF and HFmrEF. AUC—area under the curve.

The associative model demonstrated good discriminative ability, with an area under the ROC curve (AUC) of 0.789 in the combined cohort. When stratified by HF phenotype, the model maintained good and comparable discrimination: AUC 0.795 (95% CI 0.756–0.834) in HFpEF and AUC 0.818 (95% CI 0.727–0.897) in HFmrEF. The difference in discriminative performance between phenotypes was not statistically significant (ΔAUC = 0.023, 95% CI −0.074–0.111; *p* = 0.598), indicating stable predictive performance of the model across both HF phenotypes (Figure 1).

**Figure 2:**
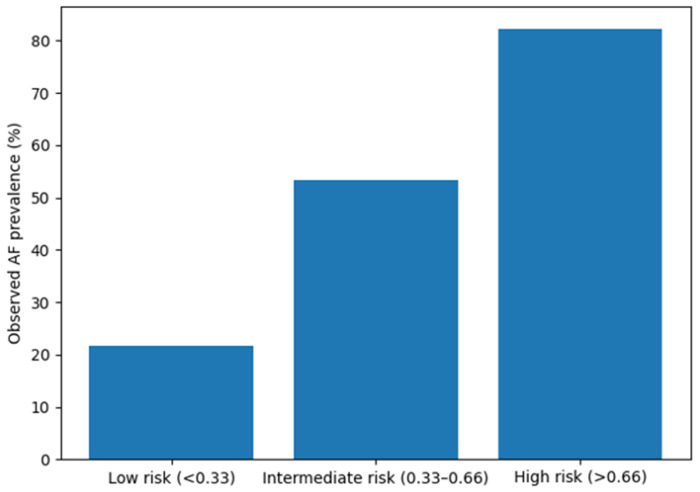
Observed AF prevalence according to predicted risk categories. AF—atrial fibrillation.

Figure 2 illustrates the observed prevalence of AF across tertiles of predicted risk derived from the multivariable model in patients with HFpEF and HFmrEF. Patients were stratified into three categories according to predicted probability: low risk (<0.33), intermediate risk (0.33–0.66), and high risk (>0.66). The observed prevalence of AF increased progressively across these groups, from 21.7% in the low-risk category to 53.3% in the intermediate-risk category and 82.3% in the high-risk category, demonstrating good discriminatory performance of our risk stratification model.

## 4. Discussion

### 4.1. Main Findings of the Study

The present study provides a comprehensive evaluation of the clinical, laboratory, and echocardiographic determinants of AF in patients with HF across different phenotypes. The main findings can be summarized as follows. First, patients with HFpEF demonstrated the broadest spectrum of factors associated with AF, including demographic characteristics, renal function parameters, hemodynamic variables, and markers of atrial remodeling. Second, in HFmrEF, the risk profile was more limited and mainly related to age, heart rate, and LA enlargement. Third, in HFrEF, the determinants of AF were predominantly associated with indices of ventricular geometry rather than atrial parameters. Finally, multivariate analysis confirmed that the factors associated with AF differ substantially between HF phenotypes, suggesting distinct pathophysiological mechanisms underlying the development of AF in these patient populations.

Although the main focus of the present study was on HFpEF and HFmrEF, HFrEF was included in the analysis as a clinically relevant comparative phenotype rather than as the primary target population. The use of HFrEF as a comparator was considered methodologically more appropriate than the inclusion of healthy controls, since both HF and AF represent complex clinical syndromes that arise in the context of aging, cardiovascular remodeling, and multimorbidity. In this setting, comparison with another HF phenotype provides a more meaningful pathophysiological reference than comparison with individuals without structural heart disease.

Importantly, this approach allowed us to demonstrate that patients with HFpEF and HFmrEF carry a substantial burden of comorbidities and thromboembolic and bleeding risk markers, as reflected by elevated CHA_2_DS_2_-VA and HAS-BLED scores. These findings suggest that patients with HFpEF and HFmrEF should not be considered clinically lower-risk populations, as their risk profile may be comparable to that observed in HFrEF. At the same time, the determinants of AF appear to vary across HF phenotypes, underscoring the importance of phenotype-specific risk assessment.

For this reason, the predictive modeling in the present study was intentionally focused on HFpEF and HFmrEF. These phenotypes remain less well-characterized with regard to AF prediction despite their rapidly increasing prevalence in contemporary clinical practice, their older age profile, and their high burden of multimorbidity. Furthermore, AF in HFpEF and HFmrEF appears to be more closely related to atrial remodeling, diastolic dysfunction, and systemic comorbidities, whereas in HFrEF the arrhythmic substrate may be influenced more strongly by ventricular remodeling and advanced systolic dysfunction. Focusing the predictive analysis on HFpEF and HFmrEF therefore addresses an important knowledge gap and may facilitate the earlier identification of patients who could benefit from intensified rhythm monitoring.

### 4.2. Interpretation of Determinants of AF in HFpEF

In the present study, HFpEF was characterized by the most complex pattern of associations with AF. Several demographic, clinical, and echocardiographic parameters were identified as independent determinants, including age, male sex, heart rate, reduced renal function, lower systolic BP, and LA enlargement, as well as the presence of moderate or severe tricuspid regurgitation. Among these variables, LA size emerged as one of the most consistent determinants, which is in line with the well-established role of atrial structural remodeling in the pathogenesis of AF.

The strong association between tricuspid regurgitation and AF observed in our cohort may reflect the combined effects of atrial dilation, pulmonary hypertension, and right-sided cardiac remodeling, which are frequently encountered in patients with HFpEF. Similarly, impaired renal function and increased heart rate may represent markers of systemic disease burden and neurohormonal activation, both of which are known contributors to arrhythmogenesis.

It should be noted that some of the identified variables, such as LA enlargement, tricuspid regurgitation, and renal dysfunction, may reflect the overall severity of cardiovascular disease and hemodynamic burden rather than acting as independent causal determinants of AF. These variables likely represent interconnected markers of structural remodeling and disease severity, highlighting the complex pathophysiological interplay between heart failure and atrial fibrillation.

Interestingly, metabolic syndrome demonstrated an inverse independent association with AF in our model. Although metabolic abnormalities are generally considered pro-arrhythmic, similar paradoxical associations have been reported in some observational studies, potentially reflecting reverse epidemiology phenomena described in cardiometabolic disease [35,36,37,38]. One possible explanation is that differences in body composition, metabolic reserve, and systemic inflammatory profiles may influence atrial structural remodeling and the electrophysiological substrate in a phenotype-dependent manner. In addition, residual confounding, competing risks, and heterogeneity within metabolic syndrome components may partly account for these unexpected associations.

### 4.3. Determinants of AF in HFmrEF

In patients with HFmrEF, the spectrum of factors associated with AF was more limited compared with HFpEF. In both univariate and multivariate analyses, LA size emerged as the only independent predictor of AF. This finding suggests that atrial structural remodeling plays a particularly central role in the development of AF in this intermediate HF phenotype.

HFmrEF is increasingly recognized as a transitional phenotype between HFpEF and HFrEF, characterized by a heterogeneous pathophysiological background. In this context, LA enlargement may represent an integrative marker reflecting the cumulative impact of chronic diastolic dysfunction, elevated filling pressures, and systemic comorbidity burden. The strong association between atrial size and AF observed in this subgroup supports the concept that atrial remodeling is a key determinant of arrhythmogenesis in patients with HFmrEF.

### 4.4. Determinants of AF in HFrEF

In contrast to HFpEF and HFmrEF, the determinants of AF in HFrEF were primarily related to indices of ventricular geometry rather than atrial parameters. In multivariate analysis, left ventricular end-diastolic volume emerged as the only independent predictor of AF. This observation suggests that ventricular remodeling and advanced systolic dysfunction may play a dominant role in the arrhythmic substrate of AF in this phenotype.

These findings are consistent with the well-established pathophysiological model of AF development in HFrEF, where ventricular dilation, elevated filling pressures, and neurohormonal activation contribute to progressive atrial structural and electrical remodeling. In this setting, ventricular parameters may indirectly reflect the severity of myocardial disease and its impact on atrial hemodynamics.

### 4.5. Comparison with Previous Studies

The results of the present study are broadly consistent with previous investigations demonstrating a strong relationship between atrial remodeling and the occurrence of AF in HF patients. LA enlargement has repeatedly been identified as one of the most robust determinants of AF across different cardiovascular populations, including patients with HFpEF and HFmrEF.

Several studies have highlighted the role of advanced age and systemic comorbidities as important contributors to AF development in patients with HF [1,39,40,41]. In particular, data from the CHART-2 registry demonstrated that advanced age is a strong independent predictor of incident AF and its progression in patients with HFpEF [42]. Our findings are consistent with these observations, showing that older age, reduced renal function, and higher heart rate were independently associated with AF in this population. These factors may collectively reflect a more advanced stage of systemic and cardiac remodeling, characterized by increased atrial pressure and volume overload, neurohormonal activation, and progressive atrial structural changes that facilitate atrial arrhythmogenesis, particularly in the HFpEF phenotype [1,39,40,41,42].

At the same time, the phenotype-specific differences observed in our analysis suggest that the mechanisms underlying AF may vary substantially across HF phenotypes. Previous studies have shown that in HFpEF and HFmrEF, the development of AF is largely driven by atrial remodeling associated with chronically elevated filling pressures, atrial dilation, and myocardial fibrosis [13,17,27,43]. In contrast, in HFrEF, the arrhythmogenic substrate may be more closely related to advanced ventricular remodeling and systolic dysfunction, which secondarily contribute to atrial dilation and electrical instability [13,28,44]. Our findings are consistent with these observations and further support the concept that the determinants of AF may differ between HF phenotypes, emphasizing the importance of considering HF phenotype when evaluating AF risk and when developing risk stratification models.

### 4.6. Clinical Implications

The identification of phenotype-specific factors associated with AF has several potential clinical implications. Early identification of patients at increased risk of AF may facilitate more targeted rhythm monitoring strategies, particularly in populations where AF may remain clinically silent for prolonged periods.

In patients with HFpEF and HFmrEF, the strong association between AF and left atrial enlargement suggests that echocardiographic assessment of atrial size may serve as a practical tool for risk stratification in routine clinical practice. Similarly, readily available clinical variables such as age, heart rate, renal function, and blood pressure may help identify patients who could benefit from intensified electrocardiographic surveillance.

Importantly, the AF associative model developed in the present study demonstrated good discriminative ability, with an AUC approaching 0.8 in the combined HFpEF/HFmrEF cohort. The ability of the model to stratify patients into distinct risk categories further supports its potential clinical utility. Patients classified as high risk may benefit from closer rhythm monitoring, prolonged ambulatory ECG monitoring, and earlier consideration of anticoagulation therapy once AF is documented.

**Figure 3:**
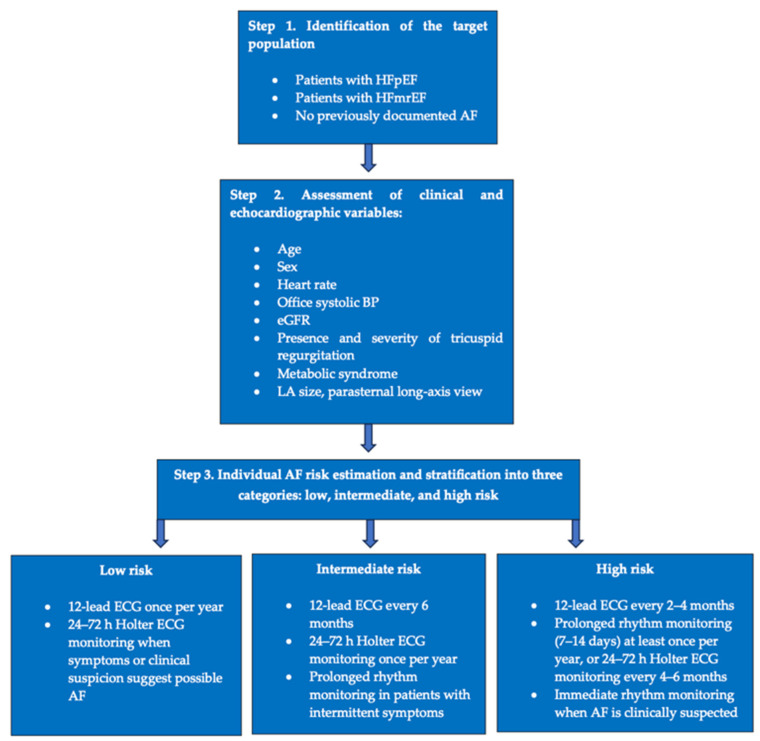
Conceptual framework for rhythm surveillance in patients with HFpEF and HFmrEF. This figure illustrates a conceptual framework derived from a multivariable model incorporating clinical and echocardiographic parameters associated with AF in the studied population. Based on the estimated probability of AF, patients are categorized into three illustrative risk groups (low, intermediate, and high risk), reflecting different potential levels of rhythm monitoring intensity. This framework is intended to summarize the identified associations and provide a basis for hypothesis generation and future research. It is not intended for direct clinical application, and the proposed stratification requires internal and external validation before any potential use in routine practice.

Based on the developed multivariable model, a conceptual framework for AF risk stratification in patients with HFpEF and HFmrEF was constructed (Figure 3). The model demonstrated good discriminative performance (AUC = 0.789) and m classification of patients into three risk categories: low, intermediate, and high risk. The proposed surveillance intervals represent a pragmatic protocol aligned with the current recommendations for AF screening and with available modalities for ambulatory ECG rhythm monitoring.

The proposed screening approach may serve as a conceptual framework for identifying patients with a higher likelihood of AF and for informing future development of risk stratification strategies. It should be considered exploratory and hypothesis-generating, as it has not undergone internal or external validation and is not intended for immediate clinical implementation.

In patients with a low a priori probability of AF, routine prolonged monitoring has limited diagnostic yield; therefore, an opportunistic screening approach based on periodic 12-lead ECG and symptom-triggered Holter monitoring appears justified.

In patients with intermediate risk, more frequent ECG assessment and periodic ambulatory ECG monitoring may be appropriate, as current recommendations allow Holter monitoring ranging from 24 h to several days.

In the high-risk group, the main objective is to reduce the time to detection of paroxysmal or subclinical AF. Prolonged ambulatory rhythm monitoring increases the probability of detecting arrhythmic episodes, while contemporary guidelines recognize extended monitoring strategies, including patch monitoring of up to 14 days, as valid screening tools. Regardless of risk category, ambulatory rhythm monitoring should be performed promptly in the presence of symptoms or clinical suspicion of AF.

### 4.7. Highlights

Phenotype-specific factors associated with AF differ across HF phenotypes.

- HFpEF suggests the most complex network of clinical and echocardiographic determinants of AF.
- LA enlargement is the most consistent determinant of AF in HFpEF and HFmrEF.
- A multivariable model combining clinical and echocardiographic parameters showed good predictive performance (AUC ~0.79).
- Risk stratification based on predicted probability effectively discriminated low-, intermediate-, and high-risk groups for AF.

### 4.8. Strengths and Limitations

Several strengths of the present study should be acknowledged. First, the analysis was based on a relatively large real-world cohort of hospitalized patients with chronic HF. Second, the study included a comprehensive evaluation of demographic, clinical, laboratory, echocardiographic, and therapeutic variables, allowing for a detailed assessment of potential determinants of AF. Third, the analysis was performed separately for different HF phenotypes, which enabled the identification of phenotype-specific determinants and improved the clinical interpretability of the findings.

However, several limitations should be considered. The retrospective single-center design may limit the generalizability of the findings. AF was identified based on electrocardiographic documentation during hospitalization or available clinical records, and rhythm monitoring was not performed systematically, which may have led to under-detection of asymptomatic or paroxysmal AF. In addition, due to the lack of longitudinal follow-up, AF was not assessed as an incident outcome; therefore, the identified variables should be interpreted as factors associated with prevalent AF rather than predictors of incident AF. The use of a complete-case analysis approach may have introduced selection bias. Although multivariable modeling was performed, the risk of overfitting cannot be excluded. Collinearity was assessed using correlation analysis, and no strong inter-variable correlations were identified; however, formal diagnostics such as variance inflation factors were not calculated. Importantly, internal validation techniques (e.g., bootstrapping or cross-validation) and formal calibration methods were not applied. As a result, the model may be subject to overfitting, and its performance may be overly optimistic. Therefore, the findings should be considered exploratory and hypothesis-generating, and external validation in independent cohorts is required before any potential clinical application can be considered. In addition, HFpEF and HFmrEF were analyzed jointly, and potential phenotype-specific differences cannot be excluded. Although HFpEF was diagnosed in accordance with the ESC guidelines, the retrospective design and reliance on available clinical data may have limited the uniform application of comprehensive diagnostic algorithms, and some degree of misclassification cannot be excluded.

## 5. Conclusions

In patients with HF, factors associated with AF differ across HF phenotypes. In the present study, HFpEF was characterized by a complex pattern of clinical and echocardiographic associations, including advanced age, male sex, higher heart rate, reduced renal function, lower systolic BP, LA enlargement, and significant tricuspid regurgitation. In contrast, AF in HFmrEF was primarily associated with LA remodeling, whereas in HFrEF, ventricular geometry emerged as the predominant correlate.

These findings support the concept that the mechanisms underlying AF vary between HF phenotypes and highlight the importance of phenotype-specific evaluation. The multivariable model developed for patients with HFpEF and HFmrEF provides an integrated representation of factors associated with AF and enables stratification across different levels of estimated probability.

However, given the cross-sectional design, lack of internal validation, and absence of formal calibration assessment, the model should be considered exploratory and hypothesis-generating. The proposed framework requires validation in independent cohorts before any potential clinical application. Future studies incorporating both internal and external validation are needed to assess the robustness and generalizability of the model.

## Figures and Tables

**Table 1:** Demographic and epidemiological characteristics of the study population according to HF phenotype.

Variable	HFpEF (*n* = 487; 69.6%)	HFmrEF (*n* = 106; 15.1%)	HFrEF (*n* = 107; 15.3%)	Total (*n* = 700)	*p*-Value
Sex, *n* (%)					
Male	179 (36.7%)	64 (60.4%)	70 (65.4%)	313 (44.7%)	<0.001
Female	308 (63.3%)	42 (39.6%)	37 (34.6%)	387 (55.3%)	
Age, median (IQR), years	73 (67–80)	73 (67–78)	74 (65–83)	74 (66–80)	0.738
Age group, *n* (%)					
<40 years	0 (0%)	0 (0%)	1 (0.9%)	2 (0.3%)	
40–49 years	0 (0%)	0 (0%)	1 (0.9%)	15 (2.1%)	
50–59 years	9 (1.8%)	3 (2.8%)	3 (2.8%)	65 (9.3%)	0.051
60–69 years	45 (9.2%)	8 (7.5%)	12 (11.2%)	177 (25.3%)	
70–79 years	120 (24.6%)	28 (26.4%)	29 (27.1%)	277 (39.6%)	
≥80 years	196 (40.2%)	50 (47.2%)	31 (29.0%)	164 (23.4%)	
COVID-19 period, *n* (%)					
Before the pandemic	210 (43.1%)	54 (50.9%)	40 (37.4%)	304 (43.4%)	0.132
After the pandemic	277 (56.9%)	52 (49.1%)	67 (62.6%)	396 (56.6%)	

HFpEF—heart failure with preserved ejection fraction; HFmrEF—heart failure with mildly reduced ejection fraction; HFrEF—heart failure with reduced ejection fraction; IQR—interquartile range.

**Table 2:** Clinical characteristics of the study population according to HF phenotype.

Variable	HFpEF (*n* = 487; 69.6%)	HFmrEF (*n* = 106; 15.1%)	HFrEF (*n* = 107; 15.3%)	Total (*n* = 700)	*p*-Value
HF duration before study inclusion, median (IQR), years	1 (1–2)	1 (1–4)	1 (1–4)	1 (1–3)	0.077
NYHA functional class, *n* (%)					
NYHA II	12 (2.5%)	1 (0.9%)	0 (0.0%)	13 (1.9%)	<0.001
NYHA III	474 (97.3%)	105 (99.1%)	101 (94.4%)	680 (97.1%)	
NYHA IV	1 (0.2%)	0 (0%)	6 (5.6%)	7 (1.0%)	
Heart rhythm, *n* (%)					
Sinus rhythm	249 (51.1%)	33 (31.1%)	35 (32.7%)	317 (45.3%)	
Paroxysmal AF	50 (10.3%)	10 (9.4%)	6 (5.6%)	66 (9.4%)	
Persistent AF	91 (18.7%)	27 (25.5%)	23 (21.5%)	141 (20.1%)	<0.001
Long-standing persistent AF	1 (0.2%)	0 (0%)	0 (0%)	1 (0.1%)	
Permanent AF	96 (19.7%)	36 (34.0%)	43 (40.2%)	175 (25.0%)	

HFpEF—heart failure with preserved ejection fraction; HFmrEF—heart failure with mildly reduced ejection fraction; HFrEF—heart failure with reduced ejection fraction; NYHA—New York Heart Association; AF—atrial fibrillation; IQR—interquartile range.

**Table 3:** Comorbidity profile and cardiovascular risk factors according to HF phenotype.

Variable	HFpEF (*n* = 487; 69.6%)	HFmrEF (*n* = 106; 15.1%)	HFrEF (*n* = 107; 15.3%)	Total (*n* = 700)	*p*-Value
Arterial hypertension	476 (97.7%)	102 (96.2%)	97 (90.7%)	675 (96.4%)	0.596
Dyslipidemia	163 (33.5%)	33 (31.1%)	39 (36.8%)	235 (33.6%)	0.678
Impaired glucose metabolism					
Impaired glucose tolerance/impaired fasting glucose	8 (1.6%)	1 (0.9%)	0 (0%)	9 (1.3%)	0.834
Type 2 diabetes mellitus	142 (29.2%)	34 (32.1%)	26 (24.3%)	202 (28.9%)	
Type 1 diabetes mellitus	5 (1.0%)	3 (2.8%)	1 (0.9%)	9 (1.3%)	
Metabolic syndrome	27 (5.5%)	2 (1.9%)	3 (2.8%)	32 (4.6%)	0.167
Weight disorders *					
Overweight	14 (2.9%)	1 (0.9%)	1 (0.9%)	16 (2.3%)	0.330
Obesity	78 (16.0%)	13 (12.3%)	14 (13.1%)	105 (15.0%)	
Coronary artery disease	144 (29.6%)	45 (42.5%)	43 (40.2%)	232 (30.1%)	0.009
Valvular heart disease #	468 (96.3%)	104 (98.1%)	105 (98.1%)	677 (96.9%)	0.455
Congenital heart disease	2 (0.4%)	1 (0.9%)	0 (0%)	3 (0.4%)	0.572
Chronic kidney disease	93 (19.1%)	23 (21.7%)	34 (31.8%)	150 (21.6%)	0.015
Cerebrovascular disease	105 (21.6%)	22 (20.8%)	31 (29.0%)	158 (22.6%)	0.224
Peripheral arterial disease	0 (0%)	0 (0%)	2 (1.9%)	2 (0.3%)	0.004
Prior cardiac surgery	74 (15.2%)	26 (24.5%)	33 (30.8%)	133 (19.0%)	<0.001
Thyroid disease	84 (17.3%)	17 (16.0%)	20 (18.7%)	121 (17.3%)	0.876
Chronic pulmonary disease ^	111 (22.8%)	25 (23.6%)	25 (23.4%)	161 (23.0%)	0.998
Obstructive sleep apnea	16 (3.3%)	6 (5.7%)	5 (4.7%)	27 (3.9%)	0.700
Smoking status					
Active smoker	63 (12.9%)	21 (19.8%)	19 (17.8%)	103 (14.7%)	
Former smoker	59 (12.1%)	20 (18.9%)	15 (14.0%)	94 (13.4%)	0.060

HFpEF—heart failure with preserved ejection fraction; HFmrEF—heart failure with mildly reduced ejection fraction; HFrEF—heart failure with reduced ejection fraction; * obesity—BMI ≥ 30.0 kg/m^2^, overweight—BMI 25.0–29.9 kg/m^2^; # moderate or severe valvular lesions; ^ chronic obstructive pulmonary disease and other chronic pulmonary disorders.

**Table 4:** Distribution of the CHA_2_DS_2_-VA score according to HF phenotype and cardiac rhythm.

HF Phenotype	Cardiac Rhythm	*n* (%)	CHA_2_DS_2_-VA Score, Median (IQR), Points	*p*-Value
HFpEF (≥50%)	Paroxysmal AF	50 (10.3%)	4 (3–5)	
	Persistent AF	91 (18.7%)	3 (3–5)	
	Long-standing persistent AF	1 (0.2%)	4 (4–4)	
	Permanent AF	96 (19.7%)	4 (3–5)	
HFmrEF (41–49%)	Paroxysmal AF	10 (9.4%)	3 (2.8–5)	0.568
	Persistent AF	27 (25.5%)	4 (2–5)	
	Permanent AF	36 (34.0%)	4 (3–5)	
HFrEF (≤40%)	Paroxysmal AF	6 (5.6%)	2.5 (1.5–4)	
	Persistent AF	23 (21.5%)	3 (2–5)	
	Permanent AF	43 (40.2%)	4 (3–5)	

HFpEF—heart failure with preserved ejection fraction; HFmrEF—heart failure with mildly reduced ejection fraction; HFrEF—heart failure with reduced ejection fraction; AF—atrial fibrillation; IQR—interquartile range.

**Table 5:** Distribution of the HAS-BLED score according to HF phenotype and cardiac rhythm.

HF Phenotype	Cardiac Rhythm	*n* (%)	HAS-BLED Score, Median (IQR), Points	*p*-Value
HFpEF (≥50%)	Paroxysmal AF	50 (10.3%)	2 (2–2)	
	Persistent AF	91 (18.7%)	2 (1–2)	
	Long-standing persistent AF	1 (0.2%)	1 (1–1)	
	Permanent AF	96 (19.7%)	2 (2–3)	
HFmrEF (41–49%)	Paroxysmal AF	10 (9.4%)	2 (1–2.25)	0.523
	Persistent AF	27 (25.5%)	2 (2–2)	
	Permanent AF	36 (34.0%)	2 (2–3)	
HFrEF (≤40%)	Paroxysmal AF	6 (5.6%)	1.5 (0.75–2.75)	
	Persistent AF	23 (21.5%)	2 (1–3)	
	Permanent AF	43 (40.2%)	2 (2–2)	

HFpEF—heart failure with preserved ejection fraction; HFmrEF—heart failure with mildly reduced ejection fraction; HFrEF—heart failure with reduced ejection fraction; AF—atrial fibrillation; IQR—interquartile range.

**Table 6:** Hemodynamic and anthropometric characteristics of the study population according to HF phenotype.

Variable	HFpEF (*n* = 487)	HFmrEF (*n* = 106)	HFrEF (*n* = 107)	Total (*n* = 700)	*p*-Value
Office systolic BP, median (IQR), mmHg	135 (120–150)	130 (120–150)	125 (110.5–140)	130 (120–150)	0.002
Office diastolic BP, median (IQR), mmHg	80 (75–90)	80 (75–90)	80 (70–90)	80 (75–90)	0.530
Body mass index, median (IQR), kg/m^2^	31 (28–33.75)	30 (29.25–30.75)	37 (24.5–41.7)	31 (28–35)	0.525
Heart rate, mean ± SD, bpm	78.3 ± 17.6	85.1 ± 20.4	89.3 ± 22.9	81.0 ± 19.4	<0.001

BP—blood pressure; HFpEF—heart failure with preserved ejection fraction; HFmrEF—heart failure with mildly reduced ejection fraction; HFrEF—heart failure with reduced ejection fraction; IQR—interquartile range; SD—standard deviation; bpm—beats per minute.

**Table 7:** Laboratory characteristics of the study population according to HF phenotype.

Variable	HFpEF *(n* = 487)	HFmrEF (*n* = 106)	HFrEF (*n* = 107)	Total (*n* = 700)	*p*-Value
Potassium (K), mmol/L, median (IQR)	4.00 (4.00–4.91)	4.00 (4.00–4.75)	4.00 (4.00–5.00)	4.00 (4.00–4.97)	0.691
Sodium (Na), mmol/L, median (IQR)	141 (138–143)	141 (138–143)	140 (138–143)	141 (138–143)	0.038
Hemoglobin (Hb), g/L, median (IQR)	137 (123–147)	134 (121–148)	134 (120–146)	136 (122–147)	0.673
Creatinine, µmol/L, median (IQR)	90 (74–112)	100 (81.5–131)	108 (86–156)	93 (77–117)	0.341
eGFR, mL/min/1.73 m^2^, median (IQR)	65 (50–85)	57 (43–85)	56 (34–73.3)	62 (45–84)	0.037

eGFR—estimated glomerular filtration rate calculated using the CKD-EPI equation [34]; HFpEF—heart failure with preserved ejection fraction; HFmrEF—heart failure with mildly reduced ejection fraction; HFrEF—heart failure with reduced ejection fraction; IQR—interquartile range.

**Table 8:** Echocardiographic characteristics of the study population according to heart failure phenotype.

Variable	HFpEF (*n* = 487)	HFmrEF (*n* = 106)	HFrEF (*n* = 107)	*p*-Value
LA size, parasternal long-axis view (mm), median (IQR)	42 (38–47)	45 (41.5–50)	48 (43.5–53.5)	<0.001
LA longitudinal diameter, apical four-chamber view (mm), median (IQR)	54 (50–62)	59 (53–63.5)	62 (55.25–69)	<0.001
Interventricular septal thickness, parasternal long-axis view (mm), median (IQR)	12 (11–13)	12 (11–13)	12 (11–13)	0.003
Posterior LV wall thickness, parasternal long-axis view (mm), median (IQR)	12 (11–12)	12 (11–12)	11 (10–12)	0.005
LV end-diastolic diameter, parasternal long-axis view (mm), median (IQR)	48 (44–52)	52 (46–59)	57 (51–62)	<0.001
LV end-systolic diameter, parasternal long-axis view (mm), median (IQR)	31 (28–35)	38 (32–42.5)	45 (39–52)	<0.001
LV end-diastolic volume, apical four-chamber view (mL), median (IQR)	94 (78.25–119)	116 (81–150)	137 (102–177.5)	<0.001
LV end-systolic volume, apical four-chamber view (mL), median (IQR)	35 (27–48.5)	59.5 (40–85)	83 (59.5–128.5)	<0.001
LV ejection fraction (%), Simpson method, median (IQR)	62 (57–67)	46 (42–48)	34 (28–39)	<0.001

HFpEF—heart failure with preserved ejection fraction; HFmrEF—heart failure with mildly reduced ejection fraction; HFrEF—heart failure with reduced ejection fraction; IQR—interquartile range; LA—left atrial; LV—left ventricular.

**Table 9:** Significant univariate determinants of AF in patients with HFpEF.

Variable	OR	95% CI	*p*-Value
HAS-BLED score (per 1-point increase)	1.419	1.120–1.797	0.004
CHA_2_DS_2_-VA score (per 1-point increase)	1.324	1.161–1.509	<0.001
HF duration (per 1-year increase)	1.134	1.044–1.231	0.003
Age (per 1-year increase)	1.064	1.043–1.086	<0.001
Heart rate (per 1 bpm increase)	1.022	1.011–1.033	<0.001
Serum creatinine (per 1 µmol/L increase)	1.005	1.001–1.010	0.015
Hemoglobin (per 1 g/L increase)	0.988	0.979–0.997	0.010
Office diastolic BP (per 1 mmHg increase)	0.982	0.967–0.998	0.028
Office systolic BP (per 1 mmHg increase)	0.985	0.977–0.993	<0.001
eGFR (per 1 mL/min/1.73 m^2^ increase)	0.975	0.966–0.984	<0.001
Severe tricuspid regurgitation	8.936	3.296–24.226	<0.001
Moderate tricuspid regurgitation	4.516	2.263–9.009	<0.001
Moderate mitral regurgitation	3.879	1.778–8.460	0.001
Severe mitral regurgitation	3.733	1.136–12.272	0.030
Concomitant CKD	3.097	1.332–7.199	0.009
Moderate aortic regurgitation	2.514	1.263–5.003	0.009
Mild aortic regurgitation	1.774	1.177–2.673	0.006
LA size, parasternal long-axis view (per mm increase)	1.123	1.088–1.159	<0.001
RA size, apical four-chamber view (per mm increase)	1.073	1.038–1.109	<0.001
LA longitudinal size, apical four-chamber view (per mm increase)	1.056	1.021–1.092	0.001
Mitral inflow E-wave velocity (per 1 m/s increase)	1.023	1.013–1.033	<0.001
Deceleration time of mitral E-wave (per 1-unit increase)	0.994	0.990–0.999	0.013
LV ejection fraction, Simpson method (per 1% increase)	0.964	0.940–0.988	0.003
Mineralocorticoid receptor antagonist therapy	2.204	1.282–3.790	0.004
Prior cardiac surgery	2.167	1.295–3.626	0.003
Angiotensin II receptor blocker therapy	0.649	0.446–0.944	0.024
Dihydropyridine calcium channel blocker therapy	0.599	0.414–0.868	0.007
Centrally acting antihypertensive therapy	0.495	0.264–0.929	0.029

BP—blood pressure; CHA_2_DS_2_-VA—Congestive heart failure, Hypertension, Age ≥75 years, Diabetes mellitus, Stroke/transient ischemic attack/thromboembolism, Vascular disease, Age 65–74 years; CI—confidence interval; CKD—chronic kidney disease; eGFR—estimated glomerular filtration rate calculated using the CKD-EPI equation [34]; HAS-BLED—Hypertension, Abnormal renal and/or liver function, Stroke, Bleeding history or predisposition, Labile international normalized ratio, Elderly age >65 years, Drugs and/or alcohol use; HF—heart failure; LA—left atrial; LV—left ventricular; OR—odds ratio; RA—right atrial.

**Table 10:** Independent multivariate determinants AF in patients with HFpEF.

Variable	Adjusted OR	95% CI	*p*-Value
Severe tricuspid regurgitation	6.957	2.482–19.499	<0.001
Moderate tricuspid regurgitation	4.092	1.977–8.466	<0.001
LA size, parasternal long-axis view (per mm increase)	1.114	1.054–1.177	<0.001
Age (per 1-year increase)	1.070	1.032–1.109	<0.001
Male sex	1.680	1.076–2.621	0.022
Heart rate (per 1 bpm increase)	1.026	1.012–1.039	<0.001
Metabolic syndrome	0.358	0.130–0.984	0.046
eGFR (per 1 mL/min/1.73 m^2^ increase)	0.983	0.973–0.993	0.001
Office systolic BP (per 1 mmHg increase)	0.985	0.977–0.993	<0.001

BP—blood pressure; CI—confidence interval; eGFR—estimated glomerular filtration rate calculated using the CKD-EPI equation [34]; LA—left atrial; OR—odds ratio.

**Table 11:** Significant univariate determinants of AF in patients with HFmrEF.

Variable	OR	95% CI	*p*-Value
LA size, parasternal long-axis view (per mm increase)	1.114	1.030–1.206	0.007
Age (per 1-year increase)	1.049	1.002–1.097	0.040
Heart rate (per 1 bpm increase)	1.030	1.005–1.055	0.019

CI—confidence interval; LA—left atrial; OR—odds ratio.

**Table 12:** Independent multivariate determinants of AF in patients with HFmrEF.

Variable	Adjusted OR	95% CI	*p*-Value
LA size, parasternal long-axis view (per mm increase)	1.142	1.011–1.291	0.033

CI—confidence interval; LA—left atrial; OR—odds ratio.

**Table 13:** Significant univariate determinants of AF in patients with HFrEF.

Variable	OR	95% CI	*p*-Value
Posterior LV wall thickness, parasternal long-axis view (per mm increase)	1.546	1.118–2.138	0.008
Age (per 1-year increase)	1.035	1.002–1.070	0.035
Heart rate (per 1 bpm increase)	1.032	1.009–1.056	0.006
LV end-diastolic volume, apical four-chamber view (per 1 mL increase)	0.986	0.975–0.998	0.020
LV end-systolic volume, apical four-chamber view (per 1 mL increase)	0.983	0.969–0.997	0.017
Office diastolic BP (per 1 mmHg increase)	0.952	0.917–0.987	0.009

BP—blood pressure; CI—confidence interval; OR—odds ratio; LV—left ventricular.

**Table 14:** Independent multivariate determinants of AF in patients with HFrEF.

Variable	Adjusted OR	95% CI	*p*-Value
LV end-diastolic volume, apical four-chamber view (per 1 mL increase)	0.981	0.964–0.998	0.033

CI—confidence interval; LV—left ventricular; OR—odds ratio.

## Data Availability

The data supporting the results of this study are available from Stefan Naydenov (snaydenov@gmail.com) upon reasonable request, subject to applicable ethical and privacy restrictions.

## Abbreviations

The following abbreviations are used in this manuscript:

AF	Atrial fibrillation
BP	Blood pressure
eGFR	Estimated glomerular filtration rate
ECG	Electrocardiography
ESC	European Society of Cardiology
HF	Heart failure
HFmrEF	Heart failure with mildly reduced ejection fraction
HFpEF	Heart failure with preserved ejection fraction
HFrEF	Heart failure with reduced ejection fraction
LA	Left atrial
LV	Left ventricular
LVEF	Left ventricular ejection fraction
